# Prognostic Value of Liver Kinase B1 (LKB1) in Gastric Cancer-Associated Tumor Microenvironment Immunity

**DOI:** 10.3390/biomedicines11030688

**Published:** 2023-02-23

**Authors:** Yongyi Chen, Siyu Chen, Jing Zhu, Xin Liu, Wangang Gong, Sihang Zhou, Songxiao Xu

**Affiliations:** 1 The Clinical Laboratory Department, The Cancer Hospital of the University of Chinese Academy of Sciences (Zhejiang Cancer Hospital), Hangzhou 310022, China; 2 The Key Laboratory of Zhejiang Province for Aptamers and Theranostics, Institute of Basic Medicine and Cancer (IBMC), Chinese Academy of Sciences, Hangzhou 310022, China

**Keywords:** LKB1, gastric cancer, clinical outcomes, immune checkpoint, CD3+CD8+ T cells

## Abstract

Liver kinase B1 (LKB1) is a tumor suppressor gene, the inactivation of which occurs frequently in different tumor types. However, whether LKB1 is associated with the clinical features of gastric cancer (GC) and regulating tumor immunity is unknown. In this study, we showed that LKB1 is highly expressed in the serum of healthy individuals (*n* = 176) compared to GC patients (*n* = 416) and is also associated with clinical outcomes and good survival rates in GC patients. Furthermore, genes associated with immune checkpoints and T cell activation, such as PD−1, PD−L1, CD8A, CD8B, CD28, and GZMM, were shown to be highly expressed in GC subgroups with high LKB1 expression. Compared with fresh gastric cancerous tissues, LKB1 was highly expressed in CD3+CD8+ and CD3+CD8+CD28+ T cells in fresh adjacent non-cancerous tissues. CD3+CD8+ T cells produced an IFN−γ anti−cancer immune response. Furthermore, the proportion of CD3+CD8+ T cells that expressed LKB had a positive correlation with IFN−γ expression. Moreover, GC patients with low LKB1 expression had a poor objective response rate, and worse progression-free survival and overall survival when treated with pembrolizumab. In conclusion, LKB1 may be a potential immune checkpoint in GC patients.

## 1. Introduction

Worldwide, gastric cancer (GC) is a public health issue and is the fifth most frequently diagnosed malignancy [1,2]. Although the common treatment approaches for GC, such as gastric resection, chemotherapy, radiation, and targeted therapies, are widely used in clinical practice [3], the 5−year survival rate of advanced GC is < 20%. As a result of advances in GC therapy [4,5], immunotherapy is considered an innovative approach [6] that has elucidated treatment of this disease [7]. In the era of immunotherapy, programmed cell death 1 (PD−1)/PD−ligand 1 (PD−L1) has been shown to be a biomarker for cancer diagnosis and prediction of response to immunotherapy, but is not sufficiently sensitive. Hence, it is important to identify novel predictive biomarkers in immunotherapy for GC.

Liver kinase B1 (LKB1), also known as STK11, is a serine/threonine kinase that is widely present in numerous tissues [8]. Increasing evidence has shown that LKB1 is a key tumor suppressor in multiple types of cancers, such as pancreatic cancer [9], melanoma [10,11], non−small cell lung cancer [12,13], and cervical cancer [14]. Furthermore, LKB1 is known to be involved in the regulation of immune cell functions [10]. LKB1 deficiency in T cells promotes the development of gastrointestinal polyposis [15], and also involves the IL−11−JAK/STAT3 pathway leading to gastrointestinal tumorigenesis [16]. Additionally, LKB1 plays a vital role in macrophage function by participating in processes, such as cell growth, metabolism, and polarization [17]. Recent studies have shown that loss of LKB1 may be involved in modulation of the tumor immune microenvironment [18]. Specifically, LKB1 deficiency suppresses T−cell activity by promoting proinflammatory cytokine production and neutrophil recruitment in the tumor microenvironment of patients with lung cancer [19]. A limited number of studies have clarified the association between LKB1 expression, clinical outcomes, immune contexture, and therapeutic responsiveness in GC.

In this study, we demonstrated an association between LKB1 expression and clinical outcomes in GC patients. In addition, we demonstrated that low LKB1 expression promotes an immunosuppressive microenvironment and might result in a poor prognosis among GC patients. Moreover, GC patients with high expression of LKB1 received greater benefit from pembrolizumab treatment. The prognostic value and potential role of LKB1 in tumor immunology are discussed herein and may contribute to understanding a possible mechanism underlying GC.

## 2. Materials and Methods

### 2.1. Patients and Blood Samples

This retrospective study was approved by the local Ethics Committee of Zhejiang Cancer Hospital (IRB−2021−154). We collected blood samples from 176 healthy adults and 416 GC patients between January 2015 and August 2022 and collected 26 pairs of fresh cancerous tissues and adjacent non−cancerous tissues from 26 GC patients. Patients with a history of other malignant tumors were excluded.

### 2.2. Expression of CEA, CA19−9, AFP, and LKB1

A commercial ELISA test kit (RX106671H; Ruixinbio, Quanzhou, Fujian, China) was used to evaluate the level of LKB1 expression in the plasma samples according to a standardized protocol. Briefly, corresponding plasma samples (50 μL) and standard model proteins (50 μL) were added to 96−well plates. Next, 100 μL of an HRP−labeled antibody was added and the 96−well plates were incubated for 1 h at 37 °C. Then, the plates were washed three times with washing buffer. The substrate mixture (100 μL) was added and the plates were analyzed in a microplate reader at OD 450.

Chemiluminescence was used to determine CEA (CFDA 20163402679, Siemens Healthcare Diagnostic Products., Shanghai, China), CA19−9 (CFDA 20173401781, Siemens Healthcare Diagnostic Products., Shanghai, China), and AFP expression (CFDA 20173400053, Siemens Healthcare Diagnostic Products., Shanghai, China). SPSS 19.0 software (IBM, Armonk, NY, USA) was used to determine the area under the receiver operating characteristic (ROC) curves of CEA, CA19−9, AFP, and LKB1. Based on TNM pathologic stage, FIGO stage, and HER2 expression, 416 GC patients were divided into different subgroups. A paired *t*-test was used to analyze the LKB1 expression in different subgroups of GC patients.

### 2.3. Analysis of LKB1 Expression in Immune Cells and the Association with Immune Checkpoints

Comprehensive Analysis on Multi−Omics of Immunotherapy in Pan−cancer [CAMOIP] (http://www.camoip.net/) (accessed on 1 January 2023) is a tool for analyzing expression and mutation data from The Cancer Genome Analysis (TCGA) and the immune checkpoint inhibitor (ICI)−treated projects. A standard processing pipeline was used to analyze immunogenicity and pathway enrichment in GC patients. CAMOIP was also used to analyze different immune cell expression according to LKB1 expression in GC patients.

The UCSC Xena database (https://xena.ucsc.edu/public) (accessed on 1 January 2023) is a cancer genomics data analysis platform that supports the visualization and analysis of histologic data from multiple cancer samples. The UCSC Xena database provides LKB1, T−cell immune checkpoint genes, T−cell activation, and antigen presentation of mRNA expression. A complex heatmap was made using R language (4.2.1). SPSS 19.0 software (IBM, Armonk, NY, USA) was used to analyze LKB1 expression based on quartiles. We divided the GC patients into LKB1 high and low expression groups according to the mediate value of LKB1 expression. A *t*-test was used to analyze T−cell immune checkpoint genes, T−cell activation, and antigen presentation of mRNA expression in GC patients into LKB1 high and low expression GC patient subgroups. *p* values < 0.001 were considered statistically significant.

### 2.4. Flow Cytometry

Fresh cancerous (*n* = 26) and fresh adjacent non−cancerous tissues (*n* = 26) obtained intraoperatively were ground into a tissue milling solution within 30 min. Milling solution and peripheral blood mononuclear cells (PBMCs) (1 × 10^6^ cells/mL) were collected in phosphate-buffered saline (PBS) (50μL, CR20012; Zhejiang Crenry, Zhejiang, China) supplemented with 0.5% bovine serum albumin (BSA) (Thermo Fisher, Waltham, MA, USA) and incubated with anti−human monoclonal antibodies for 15 min. The anti−human monoclonal antibodies were used, as shown in Table 1 and Supplementary Table S1 and Supplementary Figure S1: anti−LKB1 (PTG, Wuhan, Hubei, China); anti−CD68 (Biolegend, San Diego, CA, USA); anti−CD209 (Biolegend, San Diego, CA, USA); anti−CD28 (Biolegend, San Diego, CA, USA); anti−CD45/CD56/CD19 (Beckman Coulter, St. Louis, MO, USA); anti−CD45/CD4/CD8/CD3 (Beckman Coulter, St. Louis, MO, USA); anti−CD4, CD3 (Beckman Coulter, St. Louis, MO, USA); anti−CD45RO (Beckman Coulter, St. Louis, MO, USA); anti−CD8 (Beckman Coulter, St. Louis, MO, USA); anti−CD38 (Beckman Coulter, St. Louis, MO, USA); and anti−CD45RA (Beckman Coulter, St. Louis, MO, USA). A CD3/CD4/CD8/CD28/PD−1 detection kit was purchased from Raise Care (Hangzhou, China). CXP analysis software and a Beckman Coulter FC 500 flow cytometer were used to analyze the results [20,21]. A cytokine detection kit (Seager, Dalian, China) was used for cytokine detection in plasma samples from GC patients according to the manufacturer’s instructions.

### 2.5. Immune Cell Proportions in Fresh Tissue

A *t*-test was used to analyze immune cell proportions in fresh cancerous (*n* = 26) and fresh adjacent non−cancerous tissues (*n* = 26). The difference between LKB1+ and PD1+LKB1+ immune cell proportions in fresh cancerous and fresh adjacent non-cancerous tissues were analyzed using a paired *t*-test. SPSS 19.0 software (IBM, Armonk, NY, USA) was used to determine the correlation between immune cell proportions and cytokines in fresh cancerous and fresh adjacent non-cancerous tissues.

### 2.6. Tissue Immunohistochemistry (IHC)

The Biobank of Zhejiang Cancer Hospital provided the fresh cancerous (*n* = 26) and fresh adjacent non-cancerous tissues (*n* = 26) was used in this study. An IHC kit (Beijing Zhongshan Golden Bridge Biotechnology Co., Ltd., Beijing, China) was used to perform IHC in accordance with the manufacturer’s instructions. Briefly, paraffin sections were successively deparaffinized, rehydrated, and boiled for antigen retrieval. The primary LKB1 antibody (IPB0924 [dilution ratio, 1:100]; Baijia, Taizhou, Jiangsu, China) and interferon-gamma (IFN−γ) antibody (IPB0703 [dilution ratio, 1:100]; Baijia, Taizhou, Jiangsu, China) were incubated for 2 h at room temperature. The sections were washed three times with PBS. A histochemical polymer enhancer (400 μL) was added to paraffin sections for 20 min, followed by 3 washes with PBS. Next, secondary antibodies were added to the paraffin sections, and incubated for 20 min, followed by washing, DAB staining, counterstaining, and mounting.

### 2.7. Treatment Response of GC Patients to Pembrolizumab Based on LKB1 Expression

Treatment response to pembrolizumab and clinical data of GC patients undergoing immune checkpoint blockade (ICB) were obtained from a previous report [22]. SPSS 19.0 software (IBM, Armonk, NY, USA) was used to analyze LKB1 expression based on quartiles. We divided the GC patients into LKB1 high and low expression groups according to the mediate value of LKB1 expression. R language (4.2.1) was used to determine pembrolizumab treatment response of GC patients according to LKB1 expression. The expression of PD−L1 was analyzed by SPSS 19.0 software (IBM, Armonk, NY, USA) based on quartiles. GC patients were divided into PD−L1 high and low expression groups according to the mediate value. Based on LKB1 and PD−L1 expression, GC patients were stratified into LKB1 high and low within PD−L1 high and low expression subgroups, and Python was used to reflect the pembrolizumab treatment response in the subgroups. Overall survival and progression−free−survival of GC patients treated with pembrolizumab were analyzed using the Kaplan-Meier method with a log−rank test.

### 2.8. Statistical Analysis

Statistical analyses were performed using SPSS 19.0 software (IBM, Armonk, NY, USA). Patient survival was confirmed by telephone, and was analyzed using the Kaplan−Meier method with a log−rank test. *p* values < 0.05 were considered statistically significant. Furthermore, Kaplan−Meier Plotter (http://www.kmplot.com) (accessed on 1 January 2023) was used to confirm the effect of LKB1 (high vs. low expression) on the survival of GC patients. Python was used to reflect the clinical features and parameters of the GC patients.

## 3. Results

### 3.1. Association between LKB1 Expression and Clinic Features in GC Patients

**Figure 1:**
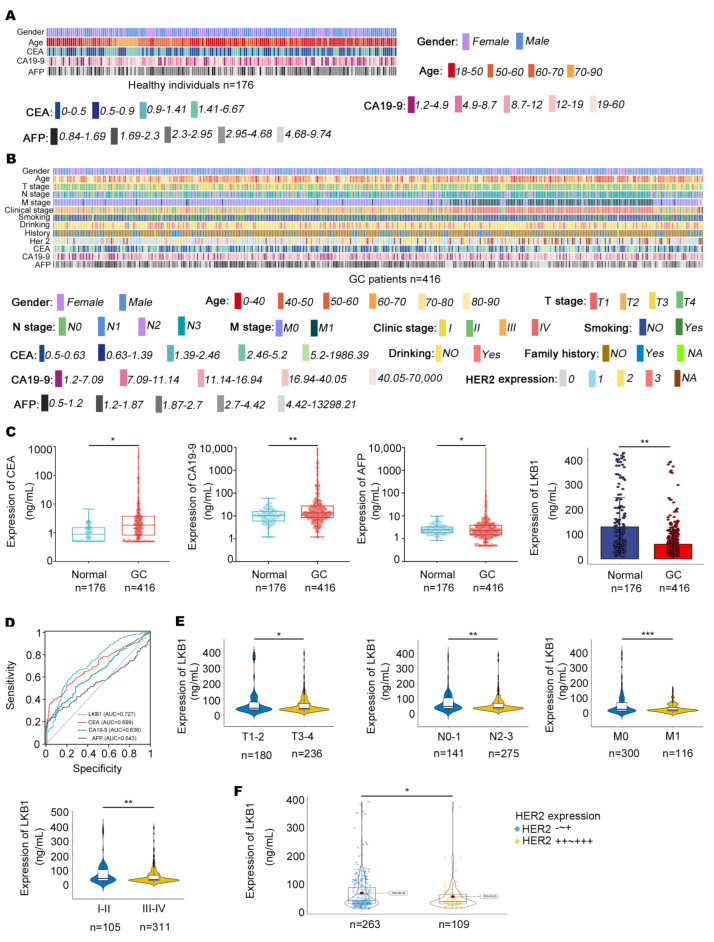
The relationships between LKB1 expression and clinical characteristics. (**A**,**B**), Clinical features in healthy individuals (*n* = 176) and GC patients (*n* = 416) were mapped using Python. (**C**), The expression of CEA, CA19−9, and AFP was measured in the serum of GC patients and healthy individuals. (**D**), Compared with CEA, CA19−9, and AFP, LKB1 had a higher sensitivity and specificity. (**E**), LKB1 expression was lower in stage N2−3, M1, and stage III−IV GC patients. (**F**), LKB1 was numerically highly expressed in HER2 negative GC patients. HER2-~+: HER2 negative GC patients. HER2++~+++: except for HER2 negative GC patients. Unpaired *t*-test, * *p* < 0.05, ** *p* < 0.01, *** *p* < 0.001.

In this study, 416 GC patients (268 males and 148 females) and 176 healthy individuals (87 males and 89 females) were included. The characteristics of healthy individuals and GC patients are summarized in Figure 1A and Table 1. The clinical outcomes of the GC patients are summarized, including CEA, CA19−9, AFP, and HER2 expression, pathologic grade, Union for International Cancer Control (UICC) tumor stage, lymph node involvement, and distant metastases (Figure 1B, Table 1 and Table 2). As shown in Figure 1C, CEA, CA19−9, and AFP were highly expressed in the serum of GC patients, while LKB1 was highly expressed in healthy individuals. Compared to CEA, CA19−9, and AFP, LKB1 had the best specificity and sensitivity (AUC = 0.727; Figure 1D). Among the clinical parameters, LKB1 was remarkably lower in stage T3−4, N2−3, M1, and stage III−IV according to UICC staging criteria (*p* < 0.05, *p* < 0.05, *p* < 0.001, and *p* < 0.01, respectively; Figure 1E and Table 2). In addition, LKB1 was numerically highly expressed in HER2 negative GC patients (Figure 1F). Thus, these results suggested that LKB1 is potentially involved in GC progression.

### 3.2. LKB1 Fosters an Immunosuppressive Microenvironment in GC Patients

**Figure 2:**
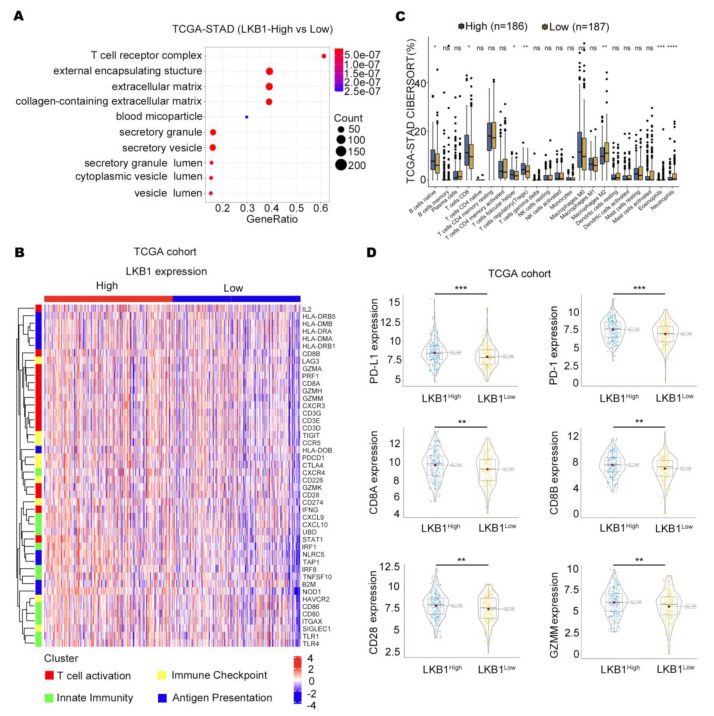
LKB1 is associated with the T−cell activation gene and immune checkpoints. (**A**) T−cell receptor complex showed the greatest difference in the LKB1 high and low expression subgroups. (**B**) CAMOIP database analysis of the differential expression of immune cells between the LKB1 high−and low−expression subgroups. (**C**) The heatmap showing the z−score normalized log−cpm values for signature immune gene sets based on LKB1 expression (*n* = 407). (**D**) PD−1, PD−L1, CD8A, CD8B, CD28, and GZMM were highly expressed in the LKB1−high GC patient subgroup. Unpaired *t*-test, ns: no significant difference, * *p* < 0.05, ** *p* < 0.01, *** *p* < 0.001, **** *p* < 0.0001.

According to the TCGA databases, the T−cell receptor complex was significantly different between GC patients with high and low LKB1 expression (Figure 2A). We further showed that the expression of T cells differed in GC patients in the high and low LKB1 expression subgroups, including CD8+ T cells, regulatory T cells, neutrophils, and eosinophils (Figure 2B). In addition, strong correlation patterns were observed between LKB1 expression and the upregulation of genes involved in T−cell immune checkpoints, T−cell activation, and antigen presentation (Figure 2C). We further stratified GC patients based on PD−1, PD−L1, CD8A, CD8B, CD28, and GZMM mRNA expression within the LKB1 high and low expression subgroups. Interestingly, the genes were all highly expressed in the LKB1−high expression subgroup (Figure 2D). LKB1, PD−1, PD−L1 expression showed limited difference in mucinous and diffuse type (Supplementary Figure S2). These data suggest that LKB1 might be associated with the T−cell activation phenotype and T−cell immune checkpoint in GC patients.

### 3.3. LKB1 Is Selectively Expressed in T (CD3+CD8+) Cell Infiltrates in GC

**Figure 3:**
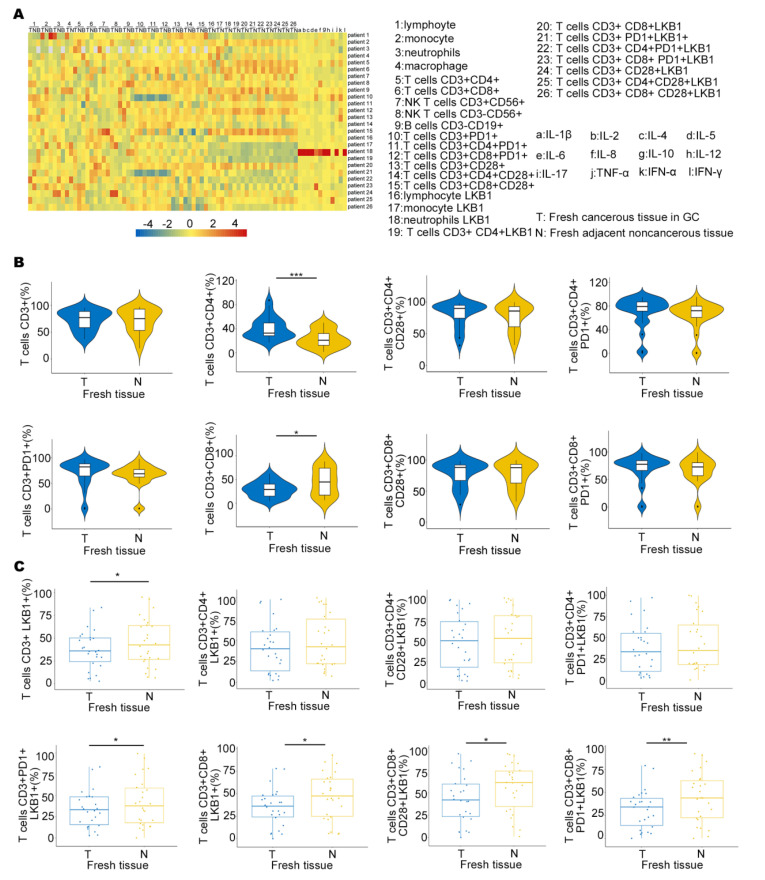
LKB1 is associated with T cell infiltration in GC patients. (**A**) The heatmap showing the immune cell proportion in peripheral blood (**B**), fresh cancerous (T) and adjacent non−cancerous tissues (N), and cytokine expression. (**B**) The ratios of different kinds of T cells in fresh cancerous (T) and adjacent non−cancerous tissues (N). (**C**) Differential proportions of T cells expressing LKB1 in fresh cancerous (T) and adjacent non−cancerous tissues (N). Paired *t*-test, * *p* < 0.05, ** *p* < 0.01, *** *p* < 0.001.

To determine LKB1 expression in the immune microenvironment, we measured the expression of six immune infiltrating cells in peripheral blood (B), fresh cancerous (T) and adjacent non−cancerous tissues (N), and 12 types of cytokine expression in GC patients (Figure 3A). We also demonstrated LKB1 expression based on the abundance of all six immune infiltration cells, including B cells, T cells, neutrophils, macrophages, and dendritic cells (Figure 3A & Supplementary Figure S3). We found that the proportion of T cells (CD3+CD8+) was higher in adjacent non−cancerous tissues (N), while the proportion of T cells (CD3+CD4+) was higher in fresh cancerous tissues (T), and other T cells showed limited differences between fresh cancerous (T) and adjacent non-cancerous tissues (N; Figure 3B). Furthermore, T cells (CD3+CD8+LKB1+, CD3+CD8+CD28+LKB1+, CD3+CD8+CD28+PD−1+LKB1+) had significantly higher ratios in fresh adjacent non−cancerous tissues (N; Figure 3C). LKB1 expression in other immune cells was similar between fresh cancerous (T) and adjacent non−cancerous tissues (N), except neutrophils (Supplementary Figure S3). Together, these results demonstrated that LKB1 might specifically be associated with the T (CD3+CD8+, CD3+CD8+CD28+) cell infiltrate microenvironment in GC patients.

### 3.4. LKB1 Is Potentially Correlated with IFN−γ Expression

**Figure 4:**
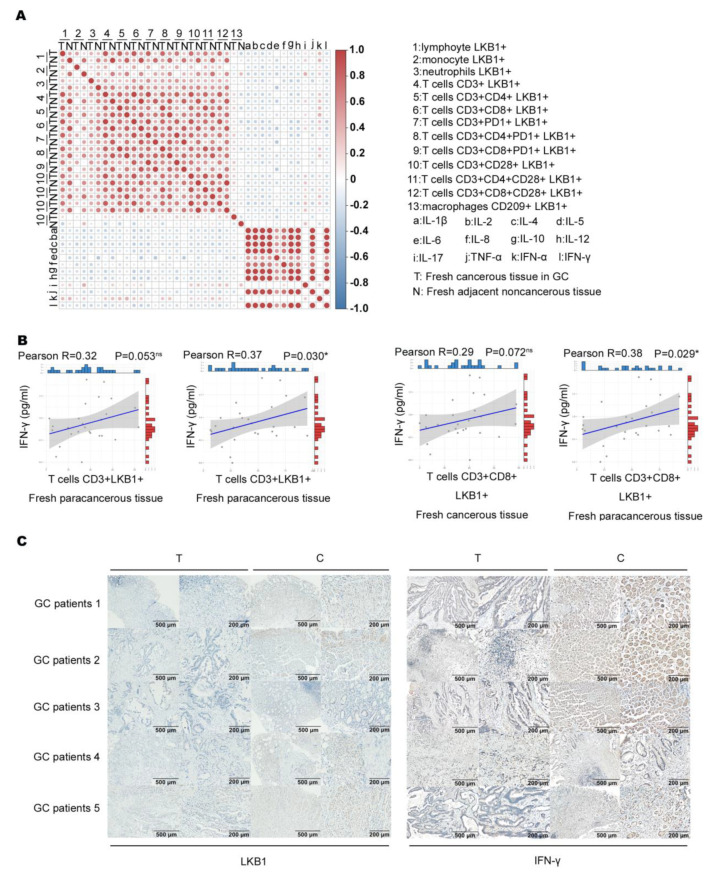
LKB1 positively correlated with IFN−γ expression. (**A**) Analysis of the association between immune cell infiltrate (LKB1+) proportion and cytokine expression. (**B**) Correlation of T cell (CD3+LKB1+) and (CD3+CD8+LKB1+) proportions with IFN−γ expression in fresh cancerous (T) and adjacent non−cancerous tissues (N). (**C**) Immunohistochemical analyses of LKB1 and IFN−γ expression in fresh cancerous (T) and adjacent non-cancerous tissues (N).

We assumed that LKB1 expressed in immune cells correlates with immune cell function. Based on the results, we analyzed the association between immune infiltration cell (LKB1+) proportion and cytokine expression (Figure 4A). A previous study showed that IFN−γ produced by T cells (CD3+CD8+) is an effective indicator for predicting clinical efficacy and survival with anti−PD−1 blockade in GC patients [23,24]. We found that only T cell (CD3+LKB1+) and (CD3+CD8+LKB1+) proportions had a slight positive correlation with IFN−γ expression in fresh cancerous (T) and adjacent non−cancerous tissues (N; Figure 4B). Next, we determined LKB1 and IFN−γ expression in the abovementioned fresh cancerous (T) and adjacent non-cancerous tissues (N), and found that LKB1 expression was significantly lower in cancerous tissues as opposed to IFN−γ expression (Figure 4C). These findings suggested that LKB1 might have a positive association with anti−tumor IFN−γ expression secreted by T cells (CD3+CD8+, CD3+CD8+CD28+).

### 3.5. LKB1 Predicts Good Responsiveness to Pembrolizumab in GC

**Figure 5:**
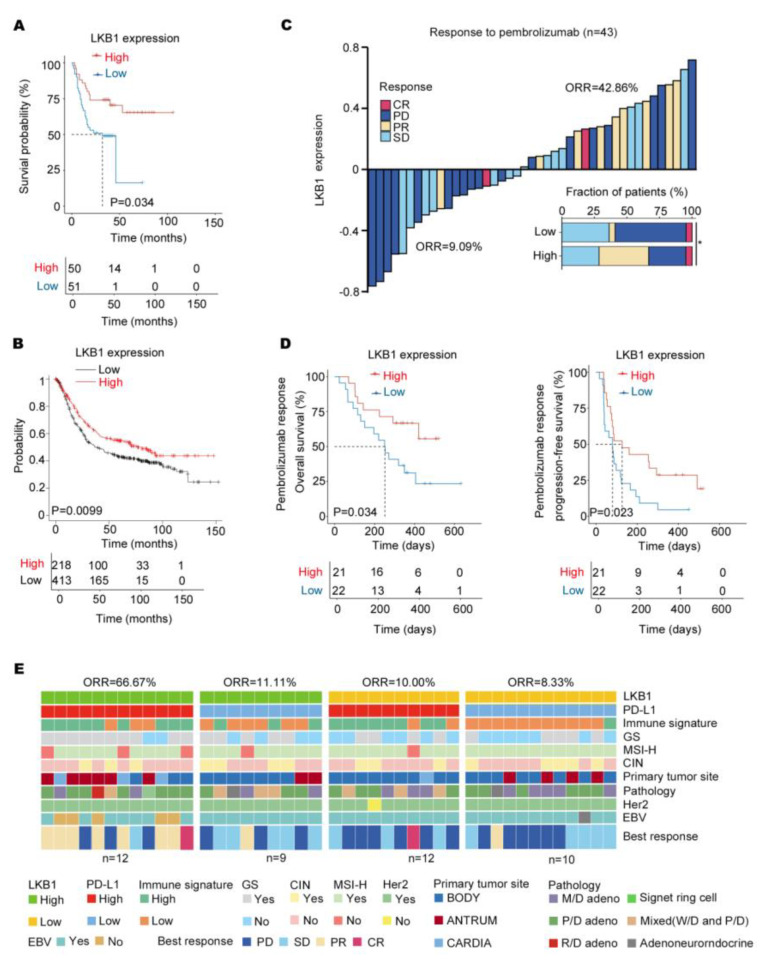
LKB1 expression predicts responsiveness to pembrolizumab in GC patients. (**A**) High levels of LKB1 expression were associated with prolonged survival in GC patients from 2015 to 2019. (**B**) The Kaplan−Meier Plotter showed that high levels of LKB1 prolonged the survival of GC patients. (**C**) The stacked bar and waterfall plots showing responsiveness to pembrolizumab in the ICB cohort (*n* = 43) based on LKB1 expression. (Pearson’s χ2 test). (**D**) Kaplan−Meier curves of progression−free survival (PFS) and overall survival (OS) in the ICB cohort (*n* = 43) based on LKB1 expression. (**E**) Based on LKB1 mRNA expression in the subgroups of GC patients with PD−L1 high and low expression, the heatmap demonstrated responsiveness to pembrolizumab and molecular parameters in the ICB cohort (*n* = 43). GS, genomically stable; MSI−H, microsatellite instability−high; EBV, EBV positive; signet ring cell: gastric signet ring cell carcinoma; ORR, objective response rate; SD, stable disease; PR, partial response; PD, progressive disease; CR, complete response; M/D adeno, moderately differentiated adenocarcinoma; P/D adeno, poorly differentiated adenocarcinoma; W/D adeno, well−differentiated adenocarcinoma; Mixed (W/D and P/D), well−differentiated adenocarcinoma and poorly differentiated adenocarcinoma.

Based on the results, LKB1 might be a potential immune checkpoint. As indicated in Figure 5A, low LKB1 expression was related to a significantly shorter overall survival based on the survival of GC patients from 2015 to 2019. Consistent with this finding, the Kaplan–Meier database (http://kmplot.com/) (accessed on 1 January 2023) also indicated that LKB1 expression significantly affected the prognosis of GC patients, with significantly lower overall survival in those patients with low LKB1 expression (Figure 5B). Next, to evaluate the predictive value of LKB1 for immunotherapy, the ICB cohort consisting of GC patients treated with pembrolizumab was analyzed (Table 3). Compared to GC patients with high LKB1 expression, the low LKB1 expression subgroup had a decreased objective response rate (ORR; Figure 5C). Furthermore, GC patients with low expression of LKB1 demonstrated a worse PFS and OS (Figure 5D). A previous study showed that the expression of PD−L1 mRNA was associated with the efficacy of pembrolizumab treatment [6]. In addition, based on LKB1 and PD−L1 expression, GC patients were stratified into LKB1 high and low within the PD−L1 high and low expression subgroups. As shown in Figure 5E, GC patients with high expression of both LKB1 and PD−L1 had the highest ORR. The correlation between PD−L1/LKB1 expression and molecular parameters in GC patients were summarized in Table 4. Taken together, LKB1 might be a potential immune checkpoint for predicting responsiveness to pembrolizumab in GC patients.

## 4. Discussion

GC is a common malignant disease and ranks as the third leading cause of cancer deaths worldwide [17,18]. In past decades, therapeutic and diagnostic strategies for GC have improved significantly [19]. Due to the lack of efficacious diagnostic markers, however, patients are often diagnosed at an advanced stage initially, with a 5−year survival rate of <20% [25,26]. Therefore, there is an urgent need to explore tumor markers with favorable specificity and sensitivity for GC diagnosis. In this study we first showed that LKB1 expression was decreased in GC serum. Compared with GC diagnostic biomarkers mainly used in clinical practice, including CEA, CA19−9, and a−1−fetoprotein (AFP), LKB1 showed the best specificity and sensitivity. Furthermore, LKB1 was associated with clinical features of GC patients, such as grade, invasion depth, TNM stage, UICC stage, and vital status. These results suggested that LKB1 serves as a tumor suppressor gene and suppress GC progression.

LKB1 was originally identified in 1997, and is also known as STK11 [27]. LKB1 is an important human tumor suppressor gene, and in non−small cell lung cancer (NSCLC) patients LKB1 mutations or genomic loss frequently co−occur with KRAS alterations. This combination results in a highly aggressive phenotype and reduced survival rate [28,29,30]. Most of the reports involving LKB1 have mainly concentrated on lung cancer, with limited reports on the role of LKB1 in GC. A previous study showed that LKB1 expression might be associated with a poor prognosis in GC patients [31]; however, whether LKB1 serves as a potential diagnostic marker and immunotherapeutic target has not been established. Our study showed, for the first time, that low expression of LKB1 led to inferior therapeutic responsiveness to pembrolizumab in patients with GC, suggesting that LKB1 might be a potential immunotherapeutic target.

Recently, immunotherapy has shown great advantages in the clinical treatment of GC [32,33]. PD−1/PD−L1 blockade has emerged as a novel and promising therapeutic strategy [34], emphasizing the importance of anti−tumor immunity and normalizing cytotoxic T lymphocyte (CTLs, CD8+) dysfunction in cancers [35]. In numerous cancers, CTL infiltration regulates tumor regression and is considered a positive prognostic indicator [36]. However, studies have indicated that dysfunction of CTL in infiltration leads to immune evasion and ultimately failure to attack cancer cells [37]. PD−1/PD−L1 blockade was demonstrated to eliminate established tumors via dysfunctional CTL reinvigorating anti−tumor immunity [38]. We showed that PD−1/PD−L1 was highly expressed in LKB1^high^ expressing subgroups of GC patients, suggesting that LKB1 might be associated with T−cell immune checkpoints. Furthermore, IFN−γ is a cytokine that inhibits viral replication and enhances the presentation of specific antigens released by CD8+T cells [39]. In addition, we found that T cell activation and antigen presentation genes were also highly expressed in the subgroups of GC patients, with LKB1^high^ expression, and T (CD3+CD8+LKB1+, CD3+CD8+CD28+LKB1+) cells had a positive correlation with IFN−γ expression in fresh tissues of GC patients. Thus, we inferred that LKB1 might impede T cell (CD3+CD8+, CD3+CD8+CD28+) secretion and is positively associated with anti−tumor IFN−γ expression. The underlying mechanism by which LKB1 is associated with cytotoxic T−cell activation warrants further investigation.

## Figures and Tables

**Table 1:** Comparison between GC patients and healthy individuals with clinical parameters.

Baseline Characteristics	Gastric Cancer Patients	Healthy Individuals
Number	416	176
Age, years, n(%)		
≤60	140 (33.65)	124 (70.45)
>60	276 (66.35)	52 (29.55)
P_50_ (P_25_, P_75_)	66 (57, 71)	49 (35, 62)
Sex, n(%)		
Male	287 (68.99)	88 (50.00)
Female	129 (31.01)	88 (50.00)
CEA, P_50_ (P_25_, P_75_)	1.84 (0.835, 3.865)	0.90 (0.50, 1.58)
CA19−9, P_50_ (P_25_, P_75_)	13.58 (8.185, 28.87)	10.40 (6.08, 16.23)
AFP, P_50_ (P_25_, P_75_)	2.30 (1.40, 4.02)	2.49 (1.86, 3.52)
LKB1, P_50_ (P_25_, P_75_)	36.47 (28.64, 70.87)	76.53 (43.76, 166.70)

**Table 2:** Baseline characteristics of GC patients.

Baseline Characteristics	Gastric Cancer Patients (*n* = 416)			
Smoking n(%)	Yes		No	
	164 (39.42)		252 (60.58)	
Drinking n(%)	Yes		No	
	129 (31.00)		287 (69.00)	
Family history of cancer n(%)	Yes		No	
	90 (21.63)		326 (78.37)	
Distant metastases n(%)	Yes		No	
	301 (72.36)		115 (27.64)	
LKB1 n(%)	High expression		Low expression	
	208 (50.00)		208 (50.00)	
CEA n(%)	High expression		Low expression	
	209 (50.24)		207 (49.76)	
AFP n(%)	High expression		Low expression	
	213 (51.20)		203 (48.80)	
CA19−9 n(%)	High expression		Low expression	
	207 (49.76)		209 (50.24)	
T classification n(%)	pT1	pT2	pT3	pT4
	20 (4.81)	42 (10.09)	119 (28.61)	235 (56.49)
N classification n(%)	pN0	pN1	pN2	pN3
	82 (19.71)	60 (14.43)	87 (20.91)	187 (44.95)
UICC stage n(%)	I	II	III	IV
	29 (6.97)	75 (18.03)	197 (47.36)	115 (27.64)
Her_2_ IHC n(%)	-~+	++~+++		No test
	263 (63.22)	109 (26.20)		44 (10.58)

**Table 3:** Objective gastric cancer patient response to pembrolizumab.

	All Patients (*n* = 43)		LKB1^high^ (*n* = 22)		LKB1^low^ (*n* = 21)	
Best overall response	No.	%(95%CI)	No.	%(95%CI)	No.	%(95%CI)
Objective response (CR+PR)	11	25.6% (12 to 39)	9	40.91% (18 to 63)	2	9.52% (0 to 23)
Disease Control						
CR	2	4.65% (0 to 11)	1	4.55% (0 to 14)	1	4.76% (0 to 18)
PR	9	20.9% (8 to 34)	8	36.36% (15 to 58)	1	4.76% (0 to 18)
SD	14	32.6% (18 to 47)	6	27.27% (7 to 48)	8	38.1% (15 to 61)
PD	18	41.86% (27 to 57)	6	27.27% (7 to 48)	12	57.14% (34 to 80)

CR, complete response; PD, progressive disease; PR, partial response; SD, stable disease.

**Table 4:** Association between LKB1/PD−L1 expression and molecular parameters.

Factors	LKB1^high^PD−L1^high^	LKB1^high^PD−L1^low^	LKB1^low^PD−L1^low^	LKB1^low^PD−L1^high^	*p* Value
All patients	12	9	12	10	
Immune signature					<0.0001
High	9	2	1	8	
Low	3	6	11	2	
Molecular subtype					
CIN	3	4	5	3	0.35
EBV	5	0	0	0	
GS	2	4	7	6	
MSI	3	1	0	1	
MSI status					
High	3	1	0	1	0.3
Low	9	8	12	9	
EBV status					
Positive	5	0	0	0	0.03
Negative	7	8	12	10	
Response					
PD	3	3	6	6	0.019
SD	1	5	5	3	
PR	7	1	1	0	
CR	1	0	0	1	

## Data Availability

The datasets generated during the current study are available from the corresponding author on reasonable request.

## Supplementary Materials

The following supporting information can be downloaded at: https://www.mdpi.com/article/10.3390/biomedicines11030688/s1, Figure S1: Represented images of flow cytometry results; Figure S2: LKB1, PD−1, PD−L1 expression in mucinous and diffuse gastric cancer type; Figure S3: The associated between LKB1 and other immune cell infiltration in GC patients.; Table S1: mAbs were used for flow cytometry.
