# Supplementary material for: Prognostic Value of Liver Kinase B1 (LKB1) in Gastric Cancer-Associated Tumor Microenvironment Immunity
Source: Biomedicines. 2023 Feb 23;11(3):688. doi: 10.3390/biomedicines11030688 (PMC10045062; doi:10.3390/biomedicines11030688)
Supplement: Supplementary file 1 [file biomedicines-11-00688-s001.zip › Supplementary Table S1.pdf]

Supplementary Table S1 Reagent or resource.

Reagent or resource	Identifier	Source
CD45-FITC/CD56-RD1/CD19-ECD/ CD3-PC5	Cat#6607073	Beckman Coulter
CD45-FITC/CD4-RD1/CD8-ECD/CD3-PC5	Cat#6607013	Beckman Coulter
CD4-PC5	Cat#A07752	Beckman Coulter
CD45RO-PE	Cat#A07787	Beckman Coulter
CD45RA-FITC	Cat#A07786	Beckman Coulter
CD8-PE	Cat#A07757	Beckman Coulter
CD38-FITC	Cat#A07778	Beckman Coulter
OptiLyse C lysing solution	Cat#A11895	Beckman Coulter
Coulter LH Series Diluent (PBS)	Cat#8547194	Beckman Coulter
Biocoll Separating Solution	Cat#L6155	Merck
Bovine serum albumin (BSA)	Cat#23208	Thermo Fisher
anti-LKB1 -CoraLite488	CL488-66719	PTG
anti-CD68-FITC	Cat#333806	Biolegend
anti-CD28-Alexa Fluor 700	Cat#302920	Biolegend
PE anti-CD209 (DC-SIGN)	Cat#330106	Biolegend
Cytokine detection kit	Cat#P420001	Seager
CD3/CD4/CD8/CD28/PD-1 detection kit	Cat#220813	Raise Care
