# Supplementary figures and images for: Prognostic Value of Liver Kinase B1 (LKB1) in Gastric Cancer-Associated Tumor Microenvironment Immunity
Source: Biomedicines. 2023 Feb 23;11(3):688. doi: 10.3390/biomedicines11030688 (PMC10045062; doi:10.3390/biomedicines11030688)

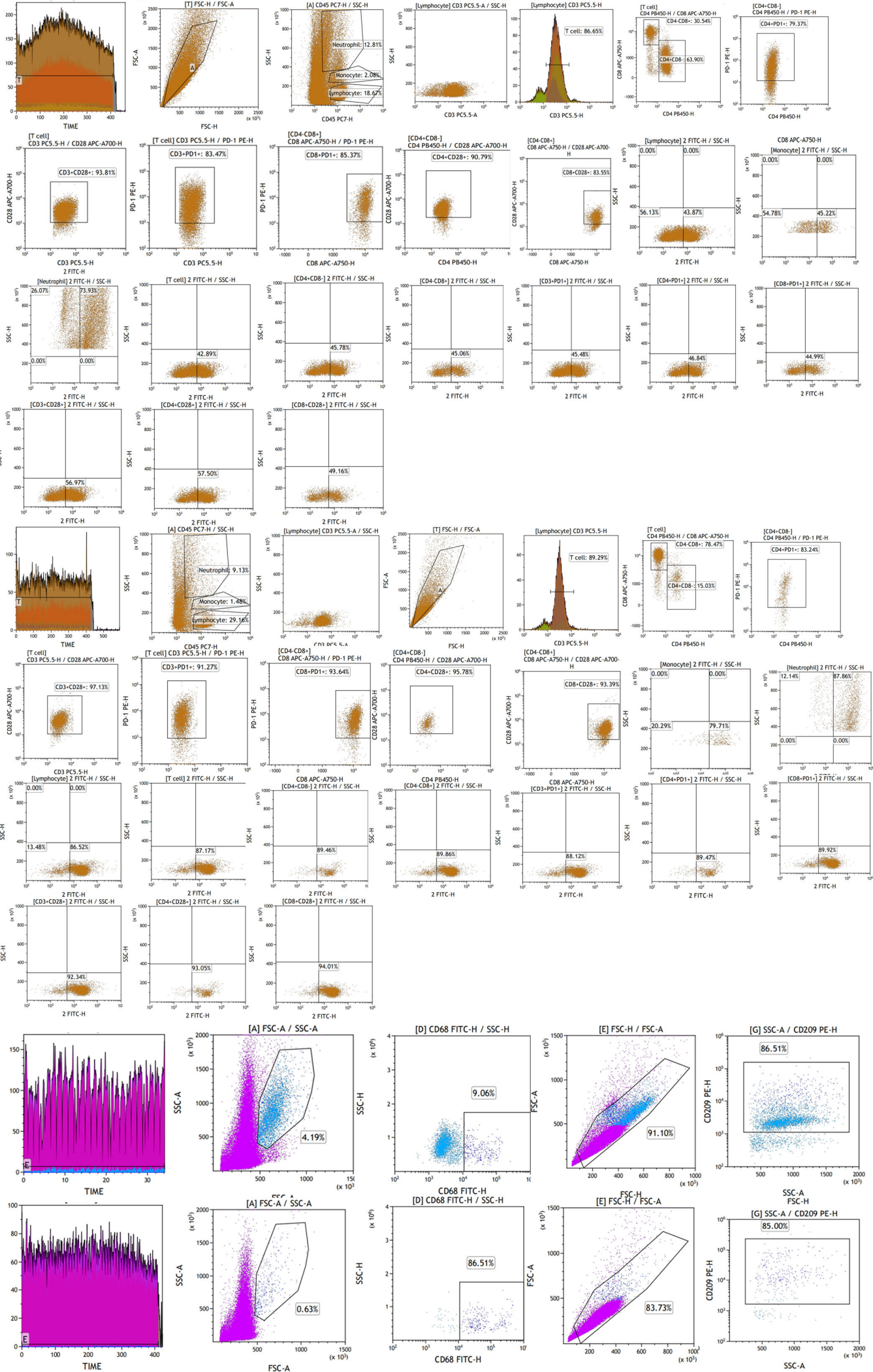

Supplement: Supplementary file 1 [file biomedicines-11-00688-s001.zip › Supplementary Figure S1.pdf]

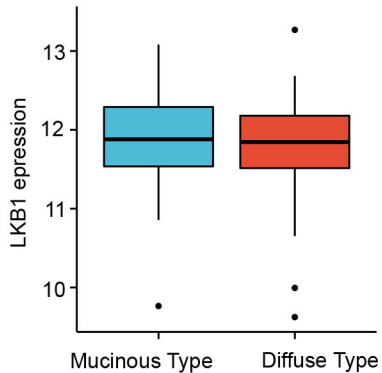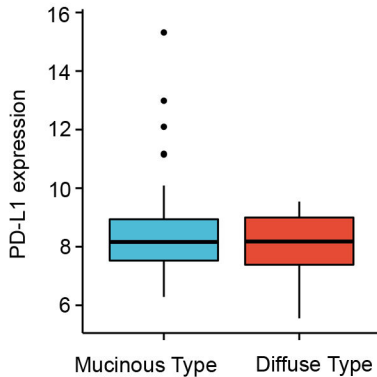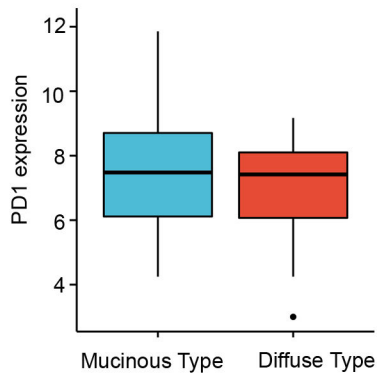

Supplement: Supplementary file 1 [file biomedicines-11-00688-s001.zip › Supplementary Figure S2.pdf]

**A**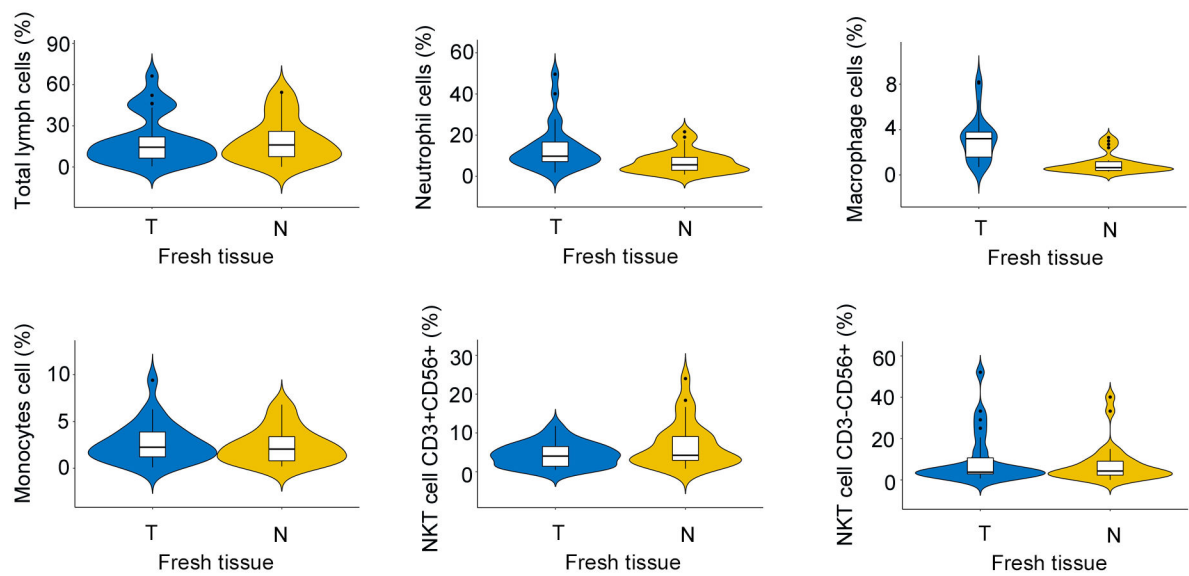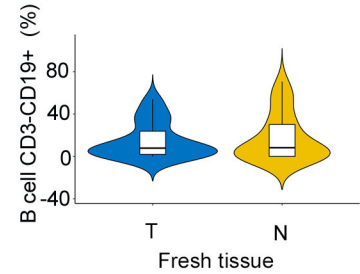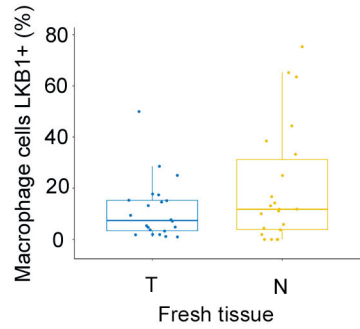**B**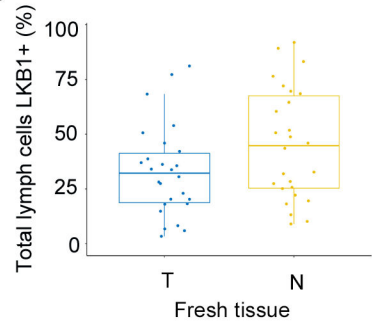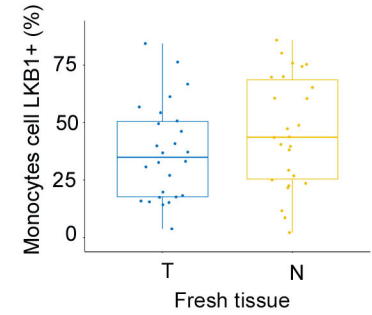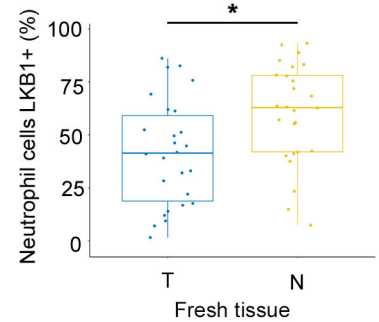

Supplement: Supplementary file 1 [file biomedicines-11-00688-s001.zip › Supplementary Figure S3.pdf]
